# Dynamics of peptidergic secretory granule transport are regulated by neuronal stimulation

**DOI:** 10.1186/1471-2202-11-32

**Published:** 2010-03-04

**Authors:** Jacqueline A Sobota, William A Mohler, Ann E Cowan, Betty A Eipper, Richard E Mains

**Affiliations:** 1 Department of Neuroscience, University of Connecticut Health Center, 263 Farmington Ave, Farmington CT 06030-3401, USA; 2 Department of Genetics and Developmental Biology, University of Connecticut Health Center, 263 Farmington Ave, Farmington CT 06030, USA; 3 Department of Molecular, Microbial and Structural Biology, University of Connecticut Health Center, 263 Farmington Ave, Farmington CT 06030, USA; 4 Center for Cell Analysis and Modeling, University of Connecticut Health Center, 263 Farmington Ave, Farmington CT 06030, USA

## Abstract

**Background:**

Peptidergic neurons store and secrete the contents of large dense core vesicles (LDCVs) from axon terminals and from dendrites. Secretion of peptides requires a highly regulated exocytotic mechanism, plus coordinated synthesis and transport of LDCVs to their sites of release. Although these trafficking events are critical to function, little is known regarding the dynamic behavior of LDCVs and the mechanisms by which their transport is regulated. Sensory neurons also package opiate receptors in peptide-containing LDCVs, which is thought to be important in pain sensation. Since peptide granules cannot be refilled locally after their contents are secreted, it is particularly important to understand how neurons support regulated release of peptides.

**Results:**

A vector encoding soluble peptidylglycine α-hydroxylating monooxygenase fused to green fluorescent protein was constructed to address these questions in cultured primary peptidergic neurons of the trigeminal ganglion using time lapse confocal microscopy. The time course of release differs with secretagogue; the secretory response to depolarization with K^+ ^is rapid and terminates within 15 minutes, while phorbol ester stimulation of secretion is maintained over a longer period. The data demonstrate fundamental differences between LDCV dynamics in axons and growth cones under basal conditions.

**Conclusions:**

Under basal conditions, LDCVs move faster away from the soma than toward the soma, but fewer LDCVs travel anterograde than retrograde. Stimulation decreased average anterograde velocity and increases granule pausing. Data from antibody uptake, quantification of enzyme secretion and appearance of pHluorin fluorescence demonstrate distributed release of peptides all along the axon, not just at terminals.

## Background

Communication within the nervous system relies on the stimulus-dependent release of neurotransmitters from vesicles. Neurons contain two vesicle classes competent for regulated exocytosis: small synaptic vesicles (SSVs) and large dense core vesicles (LDCVs, also referred to as secretory granules). Different stimuli regulate secretion from SSVs and LDCVs. Unlike neuropeptide-containing LDCVs, SSVs contain low molecular weight neurotransmitters synthesized in the cytosol [1]. Newly synthesized membrane proteins of SSVs are transported separately to the nerve terminal where they are incorporated into SSVs. Biogenesis and recycling of SSVs at the nerve terminal are closely coupled, since formation may arise from a presynaptic endosomal compartment or directly from the plasma membrane, controlling the ability of neurons to react rapidly to stimulation and sustain neurotransmitter release [2].

In contrast to synaptic vesicles, LDCVs contain peptides synthesized as inactive precursors into the endoplasmic reticulum and transported to the trans-Golgi network, to be packaged into immature LDCVs and transported down the axon [3]. These LDCVs undergo a series of maturation steps, transforming them into regulated exocytotic carriers. This biosynthetic pathway precludes local refilling, but LDCVs may engage in incomplete discharge of their contents [4]. LDCVs also deliver integral membrane proteins such as opiate receptors and ion channels to the cell surface in a regulated manner [5-9].

Little is known regarding the dynamic behavior of LDCVs and the mechanisms by which their transport is regulated in neurons. Most previous studies have utilized neuroendocrine cells such as PC12 or chromaffin cells, which preludes the analysis of events that occur during transport along long axons [4,10] Comparing the short-range motions of LDCVs in developing hippocampal neurons to their motions in neuroendocrine cells has demonstrated differences; the immobile fraction of LDCVs in neurons is smaller, and mobile LDCVs participate in exocytosis [11], while chromaffin LDCVs are usually immobile for several seconds before secretion [4].

Several lines of evidence suggest that LDCV mobility is responsive to secretagogue stimulation. In neuronally derived NS20Y cells, LDCV velocity is increased by forskolin but not by high K^+ ^[12]. At the *Drosophila *neuromuscular junction, mobilization of synaptic peptidergic LDCVs occurs following a depolarizing stimulus independent of axonal transport motors or F-actin polymerization [13]. Moreover, after synaptic peptide content has been depleted, neuropeptide stores are replenished in *en passant *terminals using retrogradely transiting LDCVs, not anterograde LDCVs [14].

The trigeminal ganglion contains primary afferent neurons which relay sensory information from craniofacial tissues. These neurons have axons but not dendrites. *In vivo*, 40-50% of these neurons contain calcitonin gene related peptide (CGRP); a subpopulation (20%) of these CGRP-positive neurons also express substance P [15]. These peptides have been implicated in the transmission of nociceptive information [16]. When dissociated trigeminal ganglion neurons are cultured in the presence of nerve growth factor (NGF), the majority show a peptidergic phenotype. NGF both supports survival of these neurons [17] and causes an increase in peptide expression [18].

LDCV dynamics in neurons have been described as either diffusive or directed, with mobilities that vary over a wide range [11]. However, these studies have not differentiated between LDCVs in transit versus those at exocytotic sites. Using primary trigeminal ganglion neurons as a model peptidergic system, we first sought to describe the dynamic behavior of LDCVs under basal conditions. We compared the behavior of LDCVs in growth cones to those in the distal portion of axons adjacent to the growth cone. We evaluated the effects of two different secretagogues on transport dynamics, including both cumulative and instantaneous parameters. Secretion was assessed by expressing a fluorescently tagged LDCV enzyme.

## Methods

### Constructs

The expression vector encoding peptidylglycine α-hydroxylating monooxygenase (PHM) has been described [19]. PAM-2-GFP was constructed in pEGFP-N2 (Clontech) by fusing in-frame the COOH-terminal of PAM (...PAPSS^976^) at an engineered SmaI site, incorporating the short intervening linker derived from pEGFP-N2 (PGIHRPVAT), followed by the N-terminus of EGFP (M^1^VSKG...). Constructs were verified by DNA sequencing.

To construct a vector encoding PHM-pHluorin, pHluorin was isolated from synaptopHluorin (gift from Dr. G. Miesenbock) by PCR. The sense primer was 5'-TATAAT *GAATTC *ATGAGCGGAAGCGGCGGGACCGGT-3' (adding EcoRI site *italics*); the antisense primer was 5'-ATATATAT *GCGGCCGC *AGATTAACCGGT TTTGTATAG-3' (adding NotI).

The PCR product and the pEGFP-N2 vector were both digested with EcoRI and NotI; the pHluorin insert was ligated into the vector, replacing EGFP (now referred to as p.pHluorin-N2). The reading frame was then corrected using the Stratagene QuickChange method of site-directed deletion mutagenesis with the sense primer 5'-CTGCTAGGAGAAAGGGAAGAATTCATGAGCGGAAG-3' and antisense primer 5'-CTTCCGCTCATGAATTCTTCCCTTTCTCCTAGCAG-3'. Next, PHM was removed from the pBluescript PAM-1 vector by digestion with XmnI and HindIII. The PHM fragment was inserted into the p.pHluorin-N2 vector digested with EcoRI, blunted with Klenow, followed by HindIII digestion, yielding PAM-1 residues 1-407 fused in reading frame with pHluorin. The construct was verified by DNA sequencing.

### Cell culture and transfections

Trigeminal ganglia were dissected from P3-P5 Sprague Dawley rat pups, digested sequentially in collagenase and trypsin, and mechanically triturated to create a single cell suspension. Cells were passed through a 70 μm nylon cell strainer, and resuspended in 3 ml L15 containing 1 mg/ml fatty acid free bovine serum albumin (BSA). This suspension was layered on top of 6 ml L15 containing 10 mg/ml BSA, centrifuged at 100 × g for 3 minutes, and the supernatant removed. This density gradient and centrifugation procedure was repeated to enrich cultures for neurons. The final cell pellet was either resuspended in L-15 growth medium (containing 10% fetal calf serum, 50 ng/ml nerve growth factor [NGF, BD Biosciences], 50 mM glucose, 250 μM ascorbate, and 8 mM glutathione) or in Rat Neuron Nucleofector solution (Amaxa). For nucleofection, the cell suspension was added to 10-12 μg DNA (for approximately 24-28 ganglia) and program G-13 was used with the Nucleofector II. Cells were resuspended in growth medium, and plated onto collagen coated 0.17 mm glass coverslips or 4 well plastic culture dishes.

### Immunocytochemistry

After 1-3 days in culture, cells were fixed in 4% formaldehyde in PBS, permeabilized in 0.075% Triton X-100 in PBS, blocked in 2 mg/ml BSA in PBS, and incubated with primary antibody overnight at 4°C. Antibodies used were rabbit anti-CGRP (MU33, 1:2000, gift from Dr. Ian Dickerson), guinea pig anti-substance P (1:1000, Neuromics), and mouse anti-βIII tubulin (TUJ1, 1:5000, Covance). Cells were rinsed 3 times with PBS, then incubated for 1 hour with secondary antibody conjugated to FITC, Cy3 (Jackson Immunoresearch), or Alexa Fluor 633 (Invitrogen). Staining was visualized using a Zeiss LSM510 confocal microscope in the Center for Cell Analysis and Modeling (University of Connecticut Health Center).

### Live cell imaging

Live neurons were imaged in L-15 without phenol red and maintained at 30°C with a Bioptechs DeltaT environmental control system. Imaging was performed on a Perkin Elmer Ultraview spinning disk confocal microscope, with image acquisition by ImageSuite software. Selective photobleaching was done using a Photonics Micropoint Mosaic digital diaphragm system. Z-stacks of 5 μm thickness (0.3 μm intervals) were acquired continuously (1.7 sec/stack, 2 × 2 binning) for 5 minutes under basal conditions, during stimulation with 1 μM phorbol 12-myristate 13-acetate (PMA) or with 50 mM KCl. This mode of imaging allowed tracking LDCVs for greater distances along the axons, moving up and down with respect to the tissue culture dish. It does place an upper limit of ~0.4 μm/sec on LDCV velocity for the tracking software to recognize the same LDCV (4 pixels in 1.7 sec). Visual inspection would have identified LDCVs moving faster than 0.5-1 μm/sec and none were seen.

To image exocytosis of PHM-pHluorin, transfected neurons were maintained at 35°C with a Zeiss stage heater, and lines scans were continuously recorded in the presence of secretagogue in line scanning mode with a Zeiss LSM 510 confocal microscope. The line selected for imaging was oriented so that it ran through the center of both an axon and a growth cone. The focal plane chosen for imaging was at the interface of the cell and coverslip. For these experiments, neurons were co-transfected with PHM-pHluorin (8 μg DNA) and pDsRed-Monomer (1 μg, Clontech) to facilitate identification of transfected cells; expression of PHM-pHluorin was verified at the conclusion of the experiment via addition of 5 mM NH_4_Cl to the medium.

### Image analysis

Maximum-intensity projections were generated from z-stacks and used for motion tracking analysis. An automated workfile was created to identify and track individual LDCVs in sequential frames using the Simple PCI motion tracking module (Compix). Identification involved passing images through a smoothing filter and setting an intensity threshold. Selected objects that appeared to be touching each other were separated, and inclusion criteria for area and roundness set identically for all image sequences. Centroid X and Y positions were calculated by the software to produce coordinates for each object in every image frame in which it was identified. Any object tracked for less than 7 frames was excluded from further analysis. Tracks were initially verified manually by checking that the center of gravity as defined by the software corresponded to the same LDCV at each timepoint throughout its trajectory. Velocity, direction, linearity, and processivity were then calculated for each LDCV being tracked. Velocity, direction and linearity for a whole run (several time points) were taken from the Simple PCI analysis. For instantaneous velocity and direction, the X-Y coordinates for each time point were used to calculate distance traveled in the time bin (hence instantaneous velocity) and the X and Y movements were used to calculate direction (0-360°). The definition of anterograde/retrograde was made using images of the axon or ending being studied.

### Antibody internalization

Neurons expressing PAM-GFP were incubated with PHM antibody (JH1761, 1:100) [19] for 15 minutes in basal medium or during stimulation with 1 mM PMA or 50 mM KCl. Cells were rinsed, fixed in 4% formaldehyde, and processed for immunocytochemistry as describe above.

### Secretion experiments and enzyme assays

Cells were rinsed 3 times with pre-warmed L-15, collections were made of basal secretion, followed by stimulation with 1 μM PMA or 50 mM KCl in L-15 (5, 15, or 30 minutes each) made by mixing L-15 with isotonic 150 mM KCl. When indicated, temperature was used as a variable and cells were kept at either room temperature or above 30°C for the duration of the experiment. Medium was centrifuged to remove non-adherent cells, and protease inhibitors added. Cells were harvested in 20 mM Na-*N*-Tris [hydroxymethyl]methyl-2-aminoethanesulfonic acid (TES), 10 mM mannitol, and 1% Triton X-100, pH 7.4 (TMT) with protease inhibitors [19], extracts were frozen and thawed three times and centrifuged to remove debris. Cell extracts and media samples were analyzed for PHM secretion by enzyme assay using ^125^I labeled α-*N*-acetyl-Tyr-Val-Gly as substrate [20]. Samples were assayed in triplicate, and reactions were carried out for 3 hours.

### Statistics

Statistical analyses were performed using SPSS (PASW 17.0).

## Results

### Use of PHM-GFP to monitor trigeminal ganglion neuron peptidergic LDCVs

**Figure 1:**
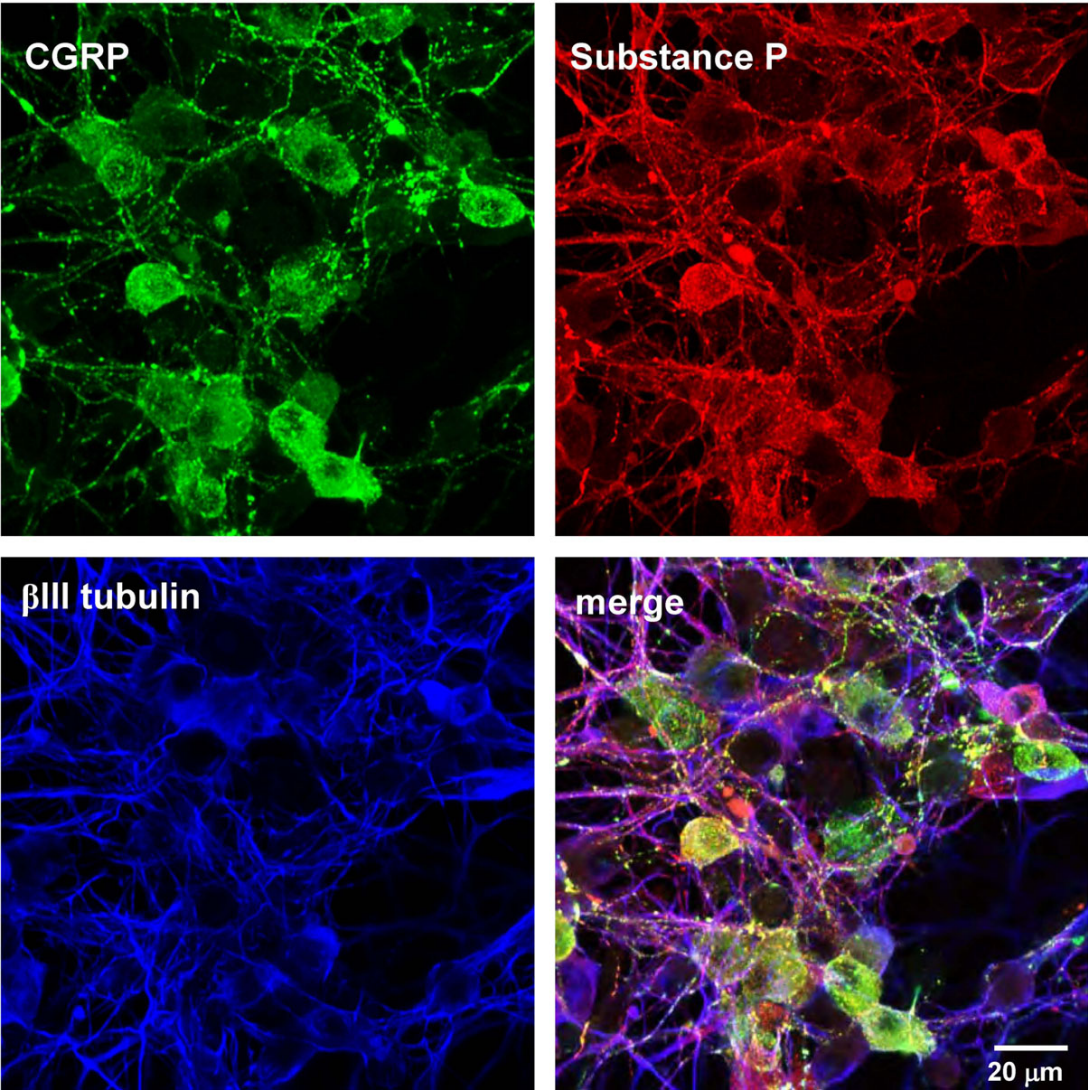
**Trigeminal ganglion neurons are peptidergic**. Trigeminal ganglia from P3-P5 rat pups were dissociated and maintained in culture for 2 days. Cells were fixed and processed for immunocytochemistry using antibodies to CGRP (green), substance P (red), and βIII tubulin (blue).

In order to investigate the dynamic behavior of LDCVs in living neurons, we utilized a primary culture system of trigeminal ganglion neurons (Figure 1). In our culture conditions, immunocytochemical analysis showed that greater than 95% of β III tubulin-positive neurons expressed either CGRP [21] or substance P or both (Figure 1). Since nociceptive neurons can be identified by their small soma size (diameter < 25 μm), all neurons analyzed met this criterion.

**Figure 2:**
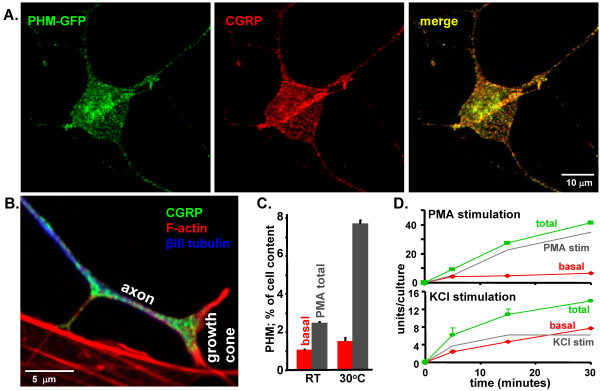
**Expression of PHM-GFP allows quantification of LDCV dynamics and secretion**. (A) Dissociated trigeminal ganglion neurons were transfected with vector encoding PHM-GFP and grown in culture for 2 days. Cells were fixed and processed for immunocytochemistry; PHM-GFP fluorescence was visualized in puncta throughout the cell soma and processes (green) along with CGRP (red). (B) A growth cone (labeled) and the preceding region of its axon are shown with immunofluorescent staining of CGRP (green) and βIII tubulin (blue); filamentous actin was visualized with a fluorescent phalloidin conjugate (red). F-actin enriched stress fibers in a non-neuronal cell are seen at the bottom of the image. (C) Duplicate cultures were analyzed at room temperature (23°C) and at 30°C; 30 minute medium collections were made under basal conditions and during stimulation with 1 μM PMA. Secretion was quantified by measuring units of PHM activity (pmol product/h) in basal and stimulated media and in cell extracts. (D) Cultures were stimulated for 5, 15, or 30 minutes at 37°C with 1 μM PMA or 50 mM KCl. Secretion was quantified by assaying units of PHM activity in basal and stimulated media; stimulated secretion was calculated by subtracting basal from total secretion (gray line); separate experiments with cultures of different densities were performed for PMA and KCl.

The soluble secretory protein PHM-GFP is efficiently targeted to LDCVs in endocrine cells [19] and was therefore expressed in trigeminal ganglion neurons. The fusion protein localized to puncta distributed throughout the cell soma and axonal processes. Simultaneous visualization of endogenous CGRP (Figure 2A) or substance P (not shown) revealed substantial colocalization. LDCVs accumulated at a higher density in growth cones (Figure 2B, green), which are the site at which regulated release is generally studied. Growth cones contained a large amount of filamentous actin (Figure 2B, red), while microtubules were more prevalent in axons (Figure 2B, blue).

Localization of PHM-GFP to the regulated secretory pathway was confirmed by quantifying basal and stimulated secretion. We chose two secretagogues with different mechanisms of action for our studies. An increase in KCl depolarizes the cells, potentially triggering action potential initiation, and opens voltage-gated calcium channels to increase secretion in a Ca^++^-dependent manner. Application of phorbol myristate acetate (PMA) activates Protein Kinase C, which phosphorylates various components of the SNARE complex as well as ion channels, causing secretion [22]. In order to select an appropriate temperature for our imaging experiments, we evaluated the effect of temperature on basal and stimulated secretion. Basal secretion of PHM-GFP was similar at 23°C (room temperature) and at 30°C (Figure 2C). Basal secretion from AtT-20 cells releases processed products from LDCVs, but is not dependent on calcium entry [23-25]. Although PMA challenge at room temperature doubled the secretion rate, raising the temperature to 30°C resulted in a further three-fold increase in PHM-GFP secretion, to 8% of cell content in 30 minutes. A similar effect of temperature on stimulated secretion was observed when pituitary cells were evaluated (Eipper & Mains, unpublished). Since regulated secretion was greatly diminished at room temperature, all of our LDCV tracking data were from cultures maintained at or slightly above 30°C.

To select the time period over which LDCV motion was evaluated, we quantified secretion of PHM-GFP for 5 to 30 minutes following application of basal medium or medium containing KCl or phorbol ester at 37°C (Figure 2D). Stimulated secretion was calculated by subtracting basal from total secretion (blue minus red = gray line). Both PMA and KCl stimulated secretion of PHM within 5 minutes. PMA-stimulated secretion persisted for the duration of the experiment, while KCl-stimulated secretion occurred primarily within 5 minutes and was completed by 15 minutes. After 30 minutes at 37°C, both PMA and KCl had stimulated the secretion of about 15% of the total cell content of PHM-GFP, a larger percentage than observed at 30°C. Based on these experiments, LDCV movement was evaluated in neurons maintained at or above 30°C and data were accumulated within the first 5 minutes after secretagogue application.

### LDCV dynamics in axons and growth cones differ under basal conditions

**Figure 3:**
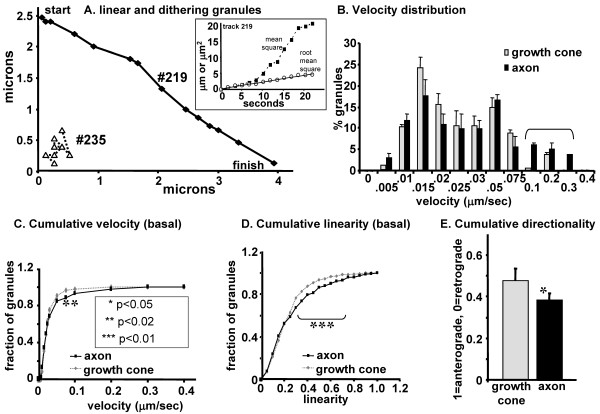
**LDCV dynamics in growth cones and axons are fundamentally different under basal conditions**. For analysis under basal conditions, neurons were kept in phenol-free L15 medium at or above 30°C. LDCVs containing PHM-GFP were tracked in growth cones and distal portions of axons. Examples of X-Y LDCV trajectories are shown; one showed rapid, directed motion (#219), and the other moved in a slow, dithering manner (#235). Velocity data for identified LDCV trajectories were pooled from 6 cells. The distribution of binned data is shown in the histogram (B), with the cumulative plot (C) emphasizing the differences between the two regions. LDCV trajectories were also analyzed to determine the distribution of linearity (D). Linearity was calculated by comparing how close the actual path was to a straight line representing the distance between the first and last point tracked. Cumulative direction was analyzed based on the mean angle of the trajectory (E) and assigned a score of 1 if anterograde or 0 if retrograde. N = 2760 LDCVs tracked.

The dynamics of LDCVs were first characterized under basal conditions. Dissociated trigeminal ganglion neurons were transfected with vector encoding PHM-GFP, and imaging experiments performed 24-48 hours later. This timing was chosen because process outgrowth occurred rapidly and growth cones were abundant. The short time made it possible to identify the cell soma associated with the growth cone chosen for imaging and distinguish anterograde from retrograde trafficking. Growth cones of small diameter neurons containing fluorescent LDCVs were identified, and the entire growth cone and the axonal segment immediately preceding it were imaged. Selective photobleaching of axonal segments was performed to facilitate LDCV tracking. Z-stacks were acquired continuously and maximum-intensity projections were generated for tracking analysis. Trajectories were generated for all tracked LDCVs based on the coordinates at each point in time; sample trajectories for two LDCVs that displayed different types of behavior are shown (Figure 3A).

LDCV #219 moved in a rapid and directed manner without changing direction, while LDCV #235 demonstrated slow, dithering motion, with frequent changes in direction. The tracking protocol was validated by manually comparing the trajectories with the actual LDCV motion in several time lapse image sets. In order to distinguish directed from diffusive motion, we used the method of Steyer and Almers to calculate diffusion constants (D) [26] for these vesicles. For the rapidly moving LDCV (#219), D is greater than 1000 × 10^-4 ^μm^2^/sec; for the dithering LDCV (#235), D = 95 × 10^-4 ^μm^2^/sec. For LDCVs diffusing freely in the cytosol, D = 10 × 10^-4 ^μm^2^/sec [26]. Polystyrene beads the size of LDCVs have comparably small diffusion coefficients when studied in viscous media [27,28]. Thus the majority of the movements we are observing are not due to diffusion.

Velocity, linearity, and direction were quantified for basal analysis of LDCVs in axons compared to growth cones. Velocity data of identified LDCV trajectories in growth cones and distal portions of axons were pooled from 6 cells each. Calculation of velocity was done as an average for the entire trajectory of each LDCV analyzed. The distribution of binned data is shown in the histogram (Figure 3B), with the cumulative plot (Figure 3C) emphasizing the differences between the two regions. For a given speed on the x-axis of the cumulative velocity plot, the associated fraction on the y-axis gives the fraction of LDCVs moving at, or below, that speed. Under basal conditions, axons contained a subpopulation of LDCVs traveling with greater velocities than those observed in growth cones (p < 0.05, n = 227; marked by asterisks). These LDCVs moved with speeds greater than 0.1 μm/sec. Statistical significance was assessed using the Kolmogorov-Smirnov test [29]. LDCVs moving faster than 0.4 μm/sec would be underestimated due to the time required for the z-stack imaging, but such fast LDCVs were not detected by visual inspection.

LDCV trajectories were further analyzed to determine the distribution of linearity. Linearity was calculated by comparing how close the actual path was to a straight line representing the distance between the first and last point, so that a maximum linearity score is 1.00. As expected, the cumulative plot showed that LDCV tracks in axons were more linear than LDCV tracks in growth cones (p < 0.025, n = 227; marked by bracket) (Figure 3D).

The net direction of LDCV movement was determined based on the average angle of each trajectory. A score of 0 was assigned for a net angle representing retrograde movement (toward the cell body), and a score of 1 for movement in the anterograde direction (directed toward the ending). Cumulative directionality scores for LDCVs in axons and growth cones did not differ significantly (Figure 3E). In growth cones, LDCVs moved in both directions with approximately equal frequencies. To our surprise, retrograde transport was slightly favored in the axon.

### An intact actin cytoskeleton is required for efficient LDCV transport

**Figure 4:**
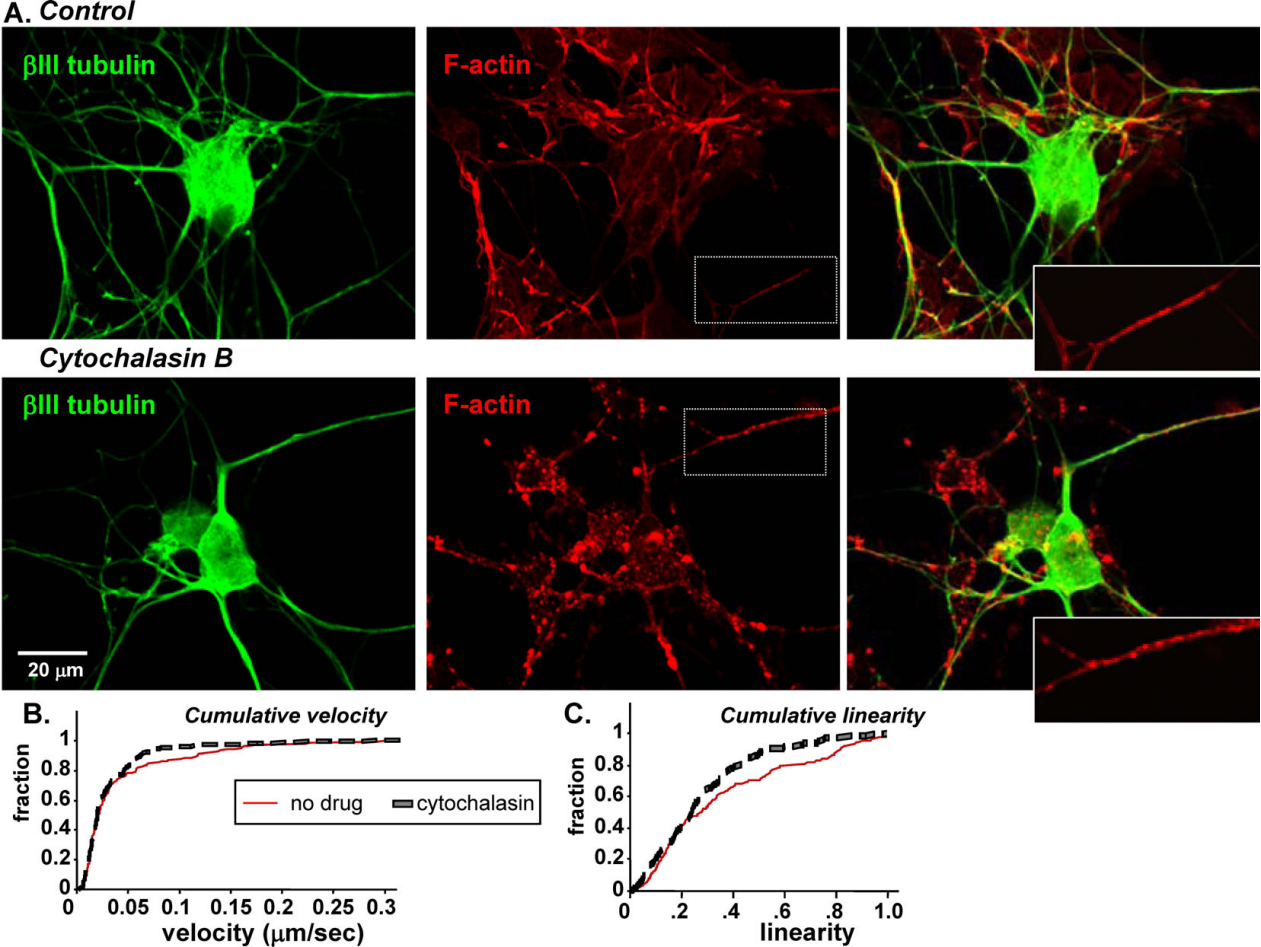
**An intact actin cytoskeleton is required for efficient LDCV transport**. Trigeminal ganglion neurons were treated with 5 μM cytochalasin B for 1 hour and then fixed and processed for immunocytochemistry (A). Filamentous actin was visualized by staining with a TRITC-phalloidin conjugate (red) and microtubules were stained with an antibody to βIII tubulin (green). Inset: phalloidin staining is shown at higher magnification to illustrate puncta that appeared in cytochalasin-treated neurons. Neurons expressing PHM-GFP were pretreated with cytochalasin B in the same manner before live imaging; cumulative velocity (B) and cumulative linearity (C) were plotted as in **Figure 3**.

The lack of directionality in growth cones as compared to axons could be related to detachment of motor proteins upon entry into growth cones, and to a prevalence of actin over microtubules. We tested whether an intact cytoskeleton were required for efficient LDCV transport by pharmacological disruption of filamentous actin with cytochalasin B. Filamentous actin was visualized using a fluorescent phalloidin conjugate, verifying that cytochalasin treatment resulted in the rearrangement of F-actin into puncta (Figure 4A, red); microtubules were unaffected (Figure 4A, green). The effect of cytochalasin treatment on LDCV dynamics was assessed; both cumulative velocity (p < 0.05) (Figure 4B) and cumulative linearity (p < 0.005) (Figure 4C) were reduced following F-actin depolymerization. Cytochalasin treatment did not have an effect on trajectory direction (not shown). The response observed may reflect a role for LDCV-associated filamentous actin in normal transport or an inhibitory effect of depolymerized actin, and further strengthens our conclusion that the movements observed are not diffusive.

### Effects of secretagogue stimulation on cumulative trajectory parameters

**Figure 5:**
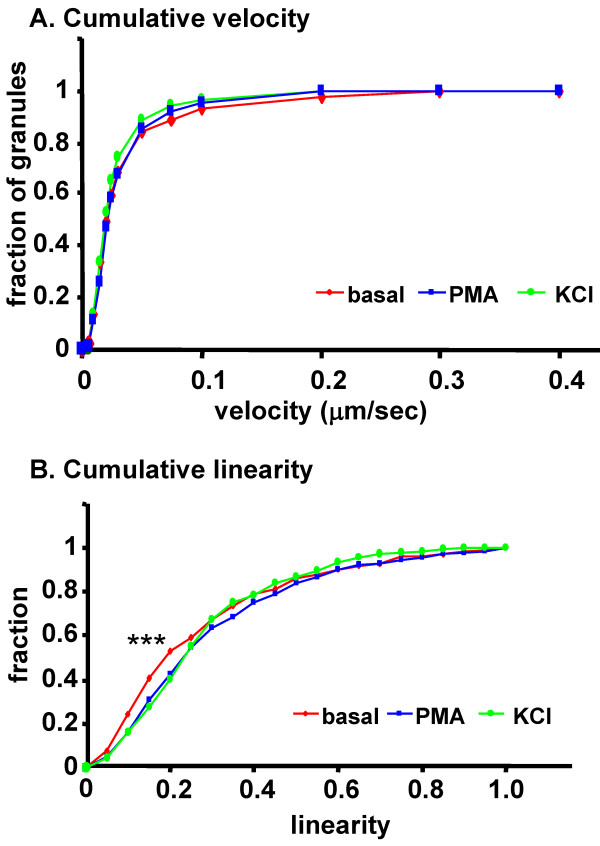
**Secretagogues increase linearity but not cumulative velocity in axons**. LDCVs containing PHM-GFP were imaged in the presence of PMA or KCl; the effects of secretagogue on dynamics were compared to behaviors observed under basal conditions. LDCV velocity and linearity were assessed in axons; data for each treatment group were pooled from 6 cells each. The distribution of cumulative velocity (A) and linearity (B) is shown for LDCVs under basal conditions and during stimulation with PMA and KCl. Both PMA and KCl had a significant effect (p < 0.01) on linearity, but not on velocity.

In addition to stimulating exocytosis, we hypothesized that secretagogue might regulate LDCV transport, perhaps by increasing directed transport to sites of exocytosis and/or initiating signaling events required to retrieve membrane components for recycling. Analysis of LDCV trafficking in axons during exposure to PMA or KCl was performed as described for basal conditions (Figure 3); data for each treatment group were pooled from 6 cells each. The distribution of cumulative velocities is shown for LDCVs during basal conditions and during stimulation with PMA and KCl (Figure 5A). Figure 5 shows that 100% of LDCVs move at speeds of 0.4 μm/sec or slower; from the plot, about half the LDCVs move at 0.025 μm/sec or slower, which may represent simple diffusion (D = 6 × 10^-4 ^μm^2^/sec). Neither drug had a significant effect on overall LDCV velocity. When linearity was analyzed (Figure 5B), both PMA (p < 0.01, n = 223) and KCl (p < 0.005) were seen to reduce the fraction of LDCVs exhibiting low linearity of motion.

### Effects of secretagogue stimulation on instantaneous trajectory parameters

**Figure 6:**
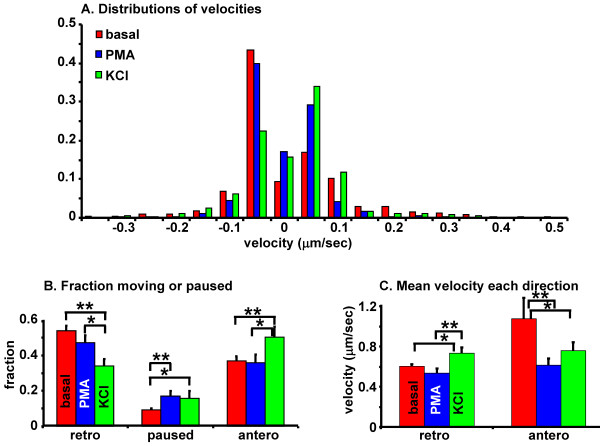
**Secretagogues differentially affect instantaneous velocity and direction**. Instantaneous velocity and direction were calculated for tracked LDCVs in neurons kept under basal conditions or exposed to PMA or KCl for 5 min. LDCVs moving in the retrograde direction were assigned a negative sign, and those moving in the anterograde direction received a positive sign (A); the distribution of binned data is shown for representative cells from each treatment group. The fraction of LDCVs stopped or moving in a given direction (B) is plotted as an average; the relationship between direction and velocity was also analyzed (C). One-way ANOVA with Bonferroni post-test (SPSS package): *, p < 0.02; **, p < 0.01.

Since the rapidly changing behavior of LDCVs was not reflected by cumulative measurements, we next assessed the effects of secretagogue on instantaneous velocity and direction. To do this, axons from three neurons were analyzed under basal conditions and after stimulation with KCl or PMA; velocity and direction were calculated for each time point (1.7 sec/stack) in the trajectory using individual LDCV coordinates. The histogram in Figure 6A shows the distribution of instantaneous velocities for each condition; within each group there was no statistically significant difference between axons. LDCVs moving in the retrograde direction were assigned a negative sign, while those moving in the anterograde direction were assigned a positive sign. LDCVs moving less than one pixel in 1.7 sec (<0.08 μm/sec) were categorized as paused.

The overall effect of secretagogue on instantaneous direction is displayed as an average for each treatment condition (Figure 6B). Under basal conditions, a larger fraction of LDCVs moved in the retrograde direction than in the anterograde direction and less than 10% of the LDCVs were classified as paused. Stimulation with either secretagogue increased the fraction of LDCVs categorized as paused, perhaps corresponding to an "immobile" fraction of LDCVs eligible for release. Stimulation with KCl also resulted in a shift from retrograde to anterograde transiting LDCVs (Figure 6B). Since LDCVs were tracked for 5 min or less, secretion was not sufficient to bias calculations of directionality (Figure 2).

Next, we analyzed the relationship between direction and velocity for each time interval (Figure 6C). LDCVs are transported away from the cell soma at a significantly faster rate than those traveling towards the cell soma under basal conditions. For those LDCVs undergoing retrograde transit, KCl increased instantaneous velocity. PMA was without effect on retrograde velocity, but reduced the average instantaneous velocity of anterograde LDCVs to half of that observed under basal conditions (Figure 6C). In contrast to its effect of LDCVs undergoing retrograde transport, KCl diminished the velocity of LDCVs traveling anterograde.

### PMA stimulation increases LDCV pausing

**Figure 7:**
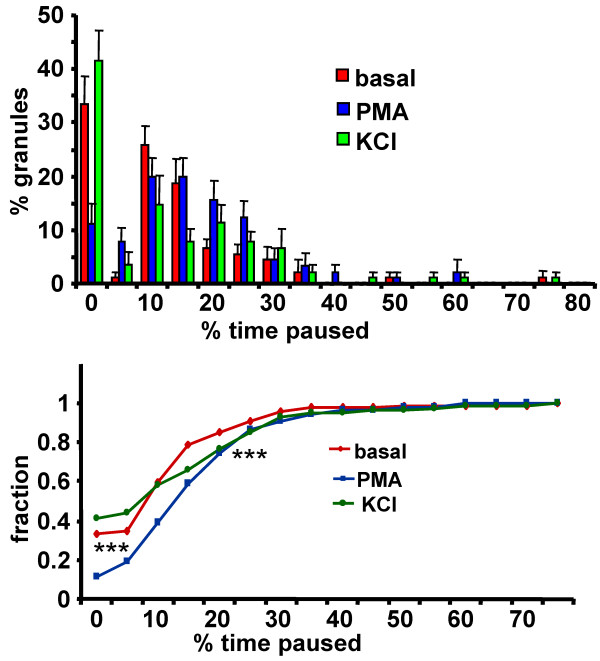
**PMA increases LDCV pausing time in axons**. The effect of secretagogue on the percentage of time that LDCVs in axons spent paused was calculated; to be categorized as 0% paused, a LDCV had to be in continuous motion. The distribution of binned data is shown.

To be fully or partially secreted, a LDCV must pause or stop, at least momentarily [26,30]. We therefore looked more closely at the pausing behavior of individual LDCVs. The effect of secretagogue on the percentage of time any given LDCV spent paused vs. moving was determined; data were plotted both as a histogram (Figure 7) and as a cumulative plot (not shown). The individual coordinates of LDCVs at each timepoint in the trajectory were used for this calculation. If the coordinates remained identical for two consecutive timepoints, the LDCV was counted as "paused." Under basal conditions, approximately 30% of the LDCVs moved continuously, with no pauses observed. Exposure to PMA decreased the fraction of LDCVs that moved continuously three-fold, to 10% (p < 0.005). In contrast, KCl was without significant effect on the percentage of time a LDCV spent paused. This result is consistent with the ability of PMA to restrict the mobility of LDCVs, which in turn correlates with an increase in priming [31].

### LDCV fusion events occur along axons and in growth cones

**Figure 8:**
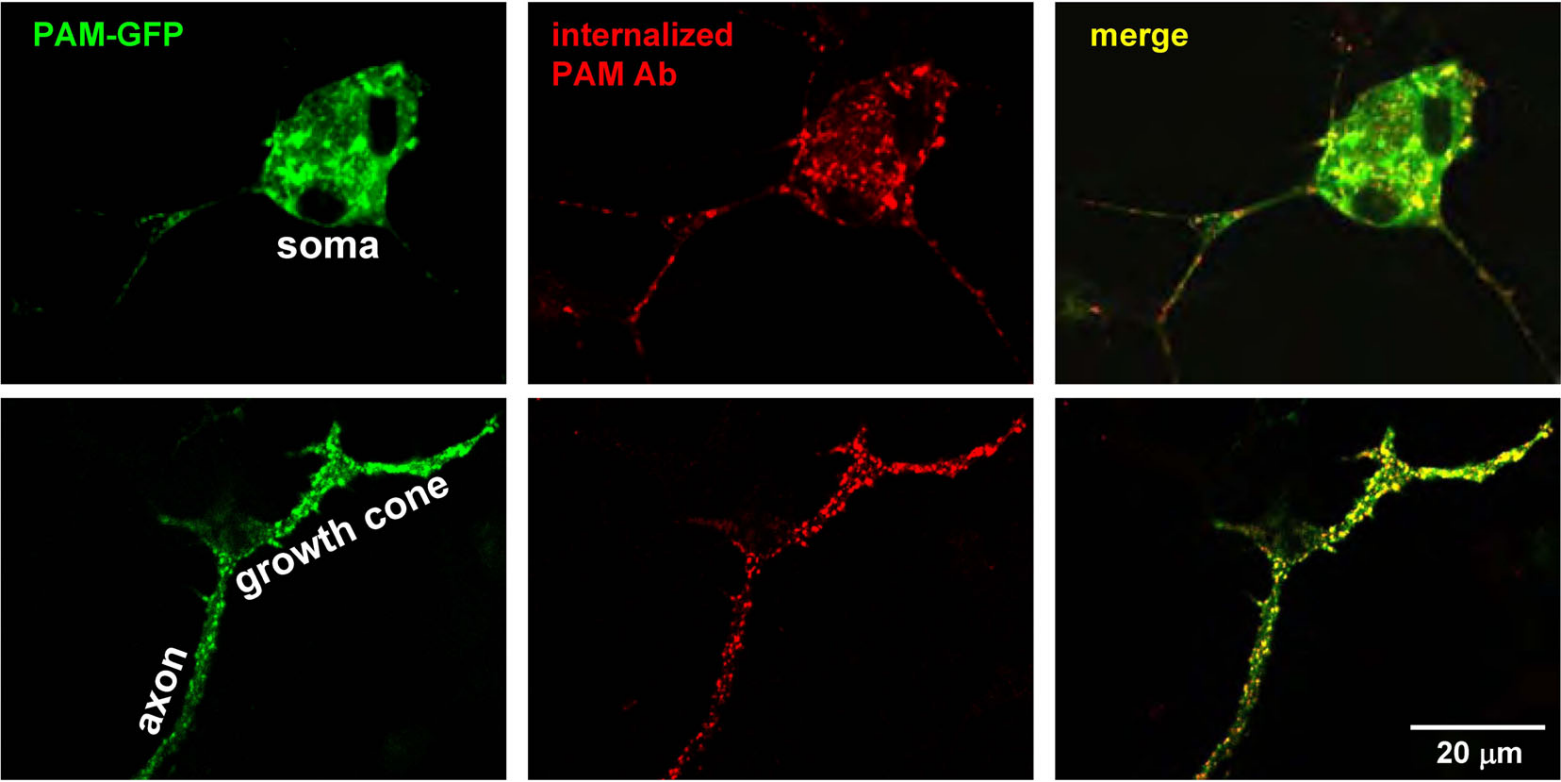
**LDCV secretion occurs throughout neurons**. Neurons were transfected with vector encoding PAM-GFP. After 2 days in culture, cells were stimulated with PMA in the presence of PAM ectodomain antibody for 15 min; internalized antibody was subsequently visualized in fixed, permeabilized cells with a Cy3 conjugated secondary antibody. Internalized PAM antibody was detected throughout the cell soma (top), along processes (bottom) and at growth cones (labeled). In the absence of secretagogue, almost no antibody uptake was observed (not shown).

More frequent stopping of LDCVs in axons is suggestive of docking and fusion events occurring along the length of the axon. To assess this possibility we used an antibody to the luminal domain of a LDCV membrane enzyme, PAM. Internalized antibody was subsequently visualized in fixed cells with a Cy3 conjugated secondary antibody. To increase the signal obtained, PAM-GFP was expressed in trigeminal neurons; PAM-GFP was visible in tubulovesicular structures in the cell soma and in vesicular structures throughout axonal processes (Figure 8, left). Under basal conditions, antibody uptake was barely detectable (not shown). When PMA was added to the cultures, uptake of PAM ectodomain antibody was readily assessed; internalized PAM antibody was present in vesicular structures throughout the cell soma (Figure 8, top), along axonal processes and at growth cones (Figure 8, bottom). Internalized antibody (red) was seen in a subset of the PAM-GFP LDCVs (yellow arrows), while other PAM-GFP LDCVs did not contain detectable antibody (green arrows). Uptake was also stimulated by KCl (not shown).

**Figure 9:**
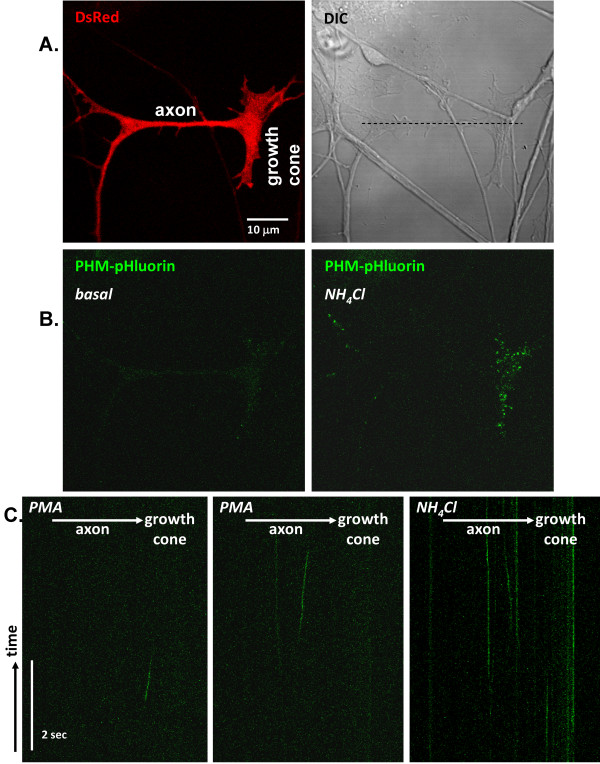
**Fusion events are detected by increases in PHM-pHluorin fluorescence**. Neurons were co-transfected with vectors encoding PHM-pHluorin and DsRed. All images in this figure are from a single microscope field of one neuron; four other neurons yielded similar results. After 2 days in culture, growth cones of neurons expressing DsRed were identified, and image frames were acquired under basal conditions (A, top left). A horizontally oriented line was placed through the center of the axon and growth cone. Scans were acquired continuously along the indicated line during PMA stimulation; two examples are shown (C, left and middle). Distance in the x dimension is represented on the x-axis, with the cell soma out of the picture to the left and the growth cone to the right. Time is represented on the y-axis; the total time for each image displayed is 6 sec. At the conclusion of the experiment, the total LDCV population was visualized by adding 5 mM NH_4_Cl to the bath solution; a frame (B, right) and line scan (C, right) are shown.

Since this approach did not permit direct visualization of fusion events in real time, we constructed a vector encoding PHM fused to pHluorin, a pH sensitive mutant of GFP [32]. Neurons were co-transfected with vectors encoding PHM-pHluorin and DsRed to enable identification of transfected cells (Figure 9A). Under basal conditions, the fluorescence of PHM-pHluorin is quenched by the acidic interior of the LDCVs, and is apparent only upon alkalinization (Figure 9B, green, right). We reasoned that LDCV release would be detectable as PHM-pHluorin was released from LDCVs that fused with the plasma membrane [32]. Diffusion of the secreted PHM-pHluorin would be expected to produce a transient increase in fluorescence at release sites.

In order to detect release of PHM-pHluorin, a horizontal line was placed through the center of the axon and growth cone (Figure 9A, right), and was continuously scanned during secretagogue stimulation. The transient appearance of green fluorescent streaks presumably correlates with individual sites of exocytosis, since the fluorescence should be visible only when the pHluorin is near neutral pH, during LDCV fusion and secretion; sample recordings during PMA stimulation are shown (Figure 9C). Each of these events occurred in a different location. The event shown in the left panel occurred in the growth cone between 1 and 2 seconds into the recording, while the event in the center panel occurred in the axon over a 2 second period later in the session. Following image acquisition, NH_4_Cl was added to the medium in order to visualize the total LDCV population containing PHM-pHluorin (Figure 9C, right). The line scan data acquired after addition of NH_4_Cl showed stabilization of PHM-pHluorin fluorescence over time, as expected; LDCVs were more prevalent in growth cones than in the axon. Based on both antibody uptake experiments and direct visualization of secretion, LDCVs are released from axons and growth cones. In five neurons recorded in detail using line scans, the vast majority of the fluorescence events (presumptive secretion) occurred in axons, not in endings/growth cones.

## Discussion

### Advantages of the trigeminal ganglion culture system for studying LDCV mobility

Our analysis of LDCV dynamics took a novel approach by utilizing primary neurons specialized to produce high levels of neuropeptides. Secretion of peptides such as CGRP and substance P from dorsal root and trigeminal ganglion neurons is an essential component of the transmission of nociceptive stimuli and contributes to peripheral inflammatory responses. In addition, these LDCVs deliver opiate receptors to the cell surface and may be involved in opiate receptor internalization as well [5]. Unlike neuroendocrine cell lines, sensory neurons such as trigeminal ganglion neurons must be equipped to transport LDCVs over long distances to reach their target tissues, both centrally and peripherally. The speed with which these neurons extend long, straight processes makes them especially useful for *in vitro *trafficking studies.

LDCV mobility is essential for sustaining the secretory response at distant sites of release. New LDCVs must come from the cell soma, the only site at which preproneuropeptides are synthesized and packaged into LDCVs. Since trigeminal ganglion neurons released 15% of their cell content during a 30 minute exposure to PMA or KCl, we hypothesized that these secretagogues would alter LDCV transport. We first compared LDCV transport in axons and growth cones. Differences were expected since long distance movements must occur in axons and secretion is generally assumed to occur at terminals.

Under basal conditions, LDCVs in axons moved with greater average velocities than LDCVs in growth cones. LDCVs moving with average velocities greater than 0.1 μm/second were seen only in axons; these rapidly moving LDCVs accounted for only ~20% of the total LDCVs observed. Trajectories were more linear in axons than in growth cones, reflecting the influence of axonal geometry. We focused our analysis on the distal segments of axons, the region immediately preceding a growth cone. To our surprise, under basal conditions, LDCVs in this region were slightly more likely to move back toward the cell soma than into the adjacent growth cone.

### Differential regulation of dynamics by secretagogues

The most significant effect of secretagogue on average LDCV dynamics was its effect on trajectory linearity. Both PMA and KCl stimulation resulted in more linear LDCV paths in axons, suggestive of motor proteins staying attached longer. The average velocity of transported LDCVs was unaffected by stimulation with KCl or PMA, consistent with the lack of effect of KCl on orphanin FQ containing LDCV velocity in NS20Y cells [12]. Average LDCV velocities in trigeminal ganglion neurons and NS20Y cells were in the same range. Average LDCV translocation rates for orphanin-GFP LDCVs in NS20Y cells were increased by forskolin, which increases intracellular cAMP by activating adenylate cyclase [12]. In PC12 cells, Ba^2+ ^stimulation increased the mobility of the subset of atrial natriuretic factor-GFP LDCVs located immediately adjacent to the plasma membrane, suggesting that the releasable pool might be expanded by making more LDCVs available to refill it [33]. Mobilization of LDCVs containing ANF-GFP, as detected by fluorescence recovery after photobleaching, was observed following depolarization, while no recovery of bouton fluorescence was observed without stimulation [13]. This liberation was proposed to facilitate replacement of secreted LDCVs, perhaps by allowing directed movement of mobilized LDCVs to other terminals to be recruited for exocytosis. In chromaffin cells, PMA limited the mobility of LDCVs in the evanescent field, and long-range motions were abolished [31]. These authors concluded that PMA increased priming, but not docking, of LDCVs; a similar phenotype was observed by overexpressing the priming factor Munc13-1 [31]. Using wide-field imaging, Shakiryanova et al. correlated a decrease in retrograde transport with an increase in synaptic neuropeptide stores at the *Drosophila *neuromuscular junction following depletion [14]. They concluded that nerve terminals do not wait passively for new LDCVs to be synthesized and transported from the soma; rather, the "mismatch" between arrival and replacement that occurs under basal conditions is the source of replenishment for the releasable pool during activity [14].

Our spinning-disk confocal microscopy approach allows us to look over a prolonged observation time and to track LDCVs that move up and down with respect to the dish surface, as they traverse the axon. Our average LDCV velocity measurements included 7 to over 30 images (10 to 50 seconds of tracking). The fact that LDCVs paused and then began to move again is not reflected in these average velocity measurements. Since LDCVs must stop at least momentarily before they can be secreted, we quantified the movements made by each LDCV from one image to the image taken 1.7 seconds later; these are designated instantaneous velocities and include all LDCVs anywhere within the region of interest. LDCVs whose centers remained in the same pixel were scored as paused. Secretion of LDCV content would result in diminution or complete loss of signal and termination of that track. Both partial secretion of a docked LDCV, which has been shown to occur [13,14], and loss of motor proteins would be scored as pausing. Although it has been proposed that stopping or pausing before secretion is very brief and might be missed [34], most investigators argue that LDCVs stop for more than 2 sec before secretion, with the majority of LDCVs stopped for over 15 sec before secretion [30].

When we analyzed the effects of PMA and KCl on instantaneous direction and velocity, significant differences were observed. KCl both increased the fraction of LDCVs undergoing anterograde transport and decreased the fraction undergoing retrograde transport. PMA did not alter the anterograde/retrograde distribution of LDCVs. Interestingly, KCl but not PMA increased instantaneous velocity in the retrograde direction, while both KCl and PMA decreased instantaneous velocity in the anterograde direction. Neuropeptides stored in the *en passant *terminals of *Drosophila *motorneurons are replenished via capture of retrogradely transiting LDCVs, not by arrival of LDCVs from the soma [14]. These differences in the effects of KCl and PMA on LDCV directionality and velocity suggest differences in the downstream targets of the signaling pathways triggered by these two secretagogues. These observations also suggest that LDCV transport is less like a unidirectional voyage, and more like sightseeing or commuting.

Most LDCVs were found to travel less than 0.2 μm/sec. Fast axonal transport is typically defined as microtubule-kinesin-dynein dependent movement at 20 mm/d or faster (0.2-4.5 μm/sec), and slower transport within axons has long been recognized to be bidirectional [35]. We found that LDCV movements faster than 0.05 μm/sec were eliminated by the actin depolymerizing drug cytochalasin (Figure 4). There is quite a large range of top speeds reported for LDCV movements, from 0.1 to over 2.0 μm/sec [11,14,36]. Some studies reporting faster velocities used differential interference contrast optics, noting that the fastest objects could be mitochondria, lysosomes or phagosomes while stationary or slowly moving objects would not be tracked at all [37]. LDCV speeds and microtubule sliding velocities are usually similar, ranging from 0.1-0.3 μm/sec [38-40]. Any LDCV moving more than 4 pixels (0.68 μm) in 1.7 sec would be considered lost (or secreted) by the tracking program (>0.4 μm/sec). Since secretion events for peptides typically take 1-2 seconds (Figure 9 and literature), the tracking software would define that as the end of a track. Given the large number of LDCVs in the axons, the small size of the LDCVs, and the extent to which the axons move in the Z-axis toward and away from the focal plane of the dish surface, we could not resolve LDCVs adequately by epifluorescence and needed the confocal quality of the images.

### Nonsynaptic localization of release sites in neurons

The vast majority of previous studies of LDCV dynamics and exocytosis utilized neuroendocrine cell lines as model systems, and nearly all were performed at room temperature. This has included chromaffin cells, which are unpolarized [26,31,36,41], and PC12 cells, which may be differentiated by NGF to extend short neuronal-like processes which accumulate LDCVs at their endings [33]. More recent work used developing hippocampal neurons to analyze motions of LDCVs containing tPA-EGFP; these were found to be highly mobile in growth cones and participate in exocytosis at this location with a slow onset after stimulation [11]. Further studies demonstrated that this fusion protein was localized to dendrites and dendritic spines [42]. LDCVs in spines were found to be immobile under both basal and stimulated conditions, and partial release of the tPA-GFP fusion protein from these LDCVs occurred over an extended period of time. One possibility is that retention of LDCVs and their peptide contents within spines may be a way to regulate the quantity of neuropeptide released, and also provide a potential mechanism to prevent depletion of peptide transmitters at postsynaptic sites [42]. In hippocampal neuronal cultures, secretion from cell bodies can be 50-times higher than from axons, based on total internal reflection microscopy [43].

Our findings that LDCVs containing PAM-GFP or PHM-pHluorin undergo fusion along axons as well as at growth cone/endings in trigeminal ganglion neurons are consistent with a role for non-synaptic peptide release. In fact, a plausible interpretation of Figures 2 and 8 together (8% secretion in 15 min yet a majority of LDCVs showing internalized antibody) is that partial release of LDCV content is more common than full fusion; with full fusion and 8% secretion, one would expect to see only a small number of yellow LDCVs after 15 min of antibody uptake. This may be particularly relevant to the normal physiology of the dorsal root and trigeminal ganglia, in which peptides such as CGRP and substance P participate in both transmission of sensory information from the periphery [44], as well as in peripheral effector functions such as neurogenic vasodilation [45]. The peripheral axons of the sensory neurons do not make synaptic contacts, yet secretion of peptides is known to contribute to local inflammatory processes. Based on our cell culture data showing 15% of PHM-GFP released in 30 min, plus PAM antibody internalization all along the axon at a level comparable to the growth cone, it seems apparent that exocytosis must occur along the length of the axon as well, perhaps serving an autoregulatory or feedback function. Whether this occurs *in vivo *has yet to be determined. It will be interesting in the future to correlate activity- dependent LDCV dynamics and secretion with physiological functions.

## Conclusions

In the peptidergic neurons of the trigeminal ganglion, LDCVs are transported away from the cell soma (anterograde) at a significantly faster rate than those traveling towards the cell soma (retrograde) under basal conditions. Anterograde LDCV velocity is slowed following depolarization by KCl or stimulation with PMA; both methods of stimulation increase granule pausing and LDCV exocytosis. Consistent with this, secretagogue stimulates the uptake of antibody that recognizes the luminal domain of a LDCV membrane protein and also stimulates the distributed appearance of a pHluorin-tagged LDCV content protein along the axon.

## Authors' contributions

JAS developed the culture system, synthesized the vectors used, acquired the images and performed the enzyme assays. WAM assisted with spinning disk confocal imaging, and AEC with line scan imaging. REM and BAE conceived of the study, assisted with performing experiments and did much of the data analysis. All authors participated in writing the manuscript, read and approved the final manuscript.
